# Development and rescue of human familial hypercholesterolaemia in a xenograft mouse model

**DOI:** 10.1038/ncomms8339

**Published:** 2015-06-17

**Authors:** Beatrice Bissig-Choisat, Lili Wang, Xavier Legras, Pradip K. Saha, Leon Chen, Peter Bell, Francis P. Pankowicz, Matthew C. Hill, Mercedes Barzi, Claudia Kettlun Leyton, Hon-Chiu Eastwood Leung, Robert L. Kruse, Ryan W. Himes, John A. Goss, James M. Wilson, Lawrence Chan, William R. Lagor, Karl-Dimiter Bissig

**Affiliations:** 1 Center for Cell and Gene Therapy, Department of Molecular and Cellular Biology, Baylor College of Medicine, Houston, Texas 77030, USA; 2 Gene Therapy Program, Department of Pathology and Laboratory Medicine, University of Pennsylvania, Philadelphia, Pennsylvania 19104, USA; 3 Division of Diabetes, Endocrinology and Metabolism, Department of Medicine, Diabetes and Endocrinology Research Center, Baylor College of Medicine, Houston, Texas 77030, USA; 4 Molecular and Cellular Biology Graduate Program, Baylor College of Medicine, Houston, Texas 77030, USA; 5 Graduate Program in Developmental Biology, Baylor College of Medicine, Houston, Texas 77030, USA; 6 Department of Pediatrics, Department of Molecular and Cellular Biology, Houston, Texas 77030, USA; 7 Dan L. Duncan Cancer Center, and Alkek Center for Molecular Discovery, Baylor College of Medicine, Houston, Texas 77030, USA; 8 Translational Biology and Molecular Medicine Graduate Program, Baylor College of Medicine, Houston, Texas 77030, USA; 9 Department of Pediatrics, Texas Children's Hospital, Houston, Texas 77030, USA; 10 Department of Surgery, Texas Children's Hospital, Houston, Texas 77030, USA; 11 Department of Molecular Physiology and Biophysics, Baylor College of Medicine, Houston, Texas 77030, USA

## Abstract

Diseases of lipid metabolism are a major cause of human morbidity, but no animal model entirely recapitulates human lipoprotein metabolism. Here we develop a xenograft mouse model using hepatocytes from a patient with familial hypercholesterolaemia caused by loss-of-function mutations in the low-density lipoprotein receptor (LDLR). Like familial hypercholesterolaemia patients, our familial hypercholesterolaemia liver chimeric mice develop hypercholesterolaemia and a 'humanized‘ serum profile, including expression of the emerging drug targets cholesteryl ester transfer protein and apolipoprotein (a), for which no genes exist in mice. We go on to replace the missing LDLR in familial hypercholesterolaemia liver chimeric mice using an adeno-associated virus 9-based gene therapy and restore normal lipoprotein profiles after administration of a single dose. Our study marks the first time a human metabolic disease is induced in an experimental animal model by human hepatocyte transplantation and treated by gene therapy. Such xenograft platforms offer the ability to validate human experimental therapies and may foster their rapid translation into the clinic.

## Abstract (web summary)

Familial hypercholesterolemia (FH) is a congenital disease associated with high plasma cholesterol levels. Here, the authors recapitulate FH in chimeric mice, in which livers are repopulated with hepatocytes from an FH patient, and successfully correct the disease using adenovirus-mediated gene therapy.

## Graphical abstract

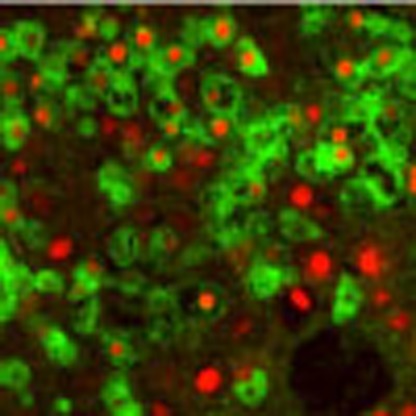

Familial hypercholesterolaemia is an autosomal dominant disorder characterized by the inability to clear low-density lipoprotein (LDL) particles from the circulation, which leads to high total plasma cholesterol levels, atherosclerosis and cardiovascular disease^1^,^2^,^3^. Familial hypercholesterolaemia is typically caused by loss-of-function mutations in the LDL receptor (LDLR)^3^,^4^,^5^, which is highly expressed in the liver, the organ primarily responsible for clearing (LDL cholesterol. Heterozygous patients usually respond to medical management, but patients with two loss-of-function alleles (compound heterozygotes and homozygous familial hypercholesterolaemia) develop severe cardiovascular disease in the first decades of life that they require more aggressive therapies, such as apheresis or liver transplantation^6^.

Although the three best-studied animal models of familial hypercholesterolaemia (the Watanabe Heritable Hyperlipidemic rabbit^7^, the rhesus macaque^8^ and the *LDLR* knockout mouse^9^) have yielded important insights into the disease, none of them fully recapitulates human lipid metabolism because the key proteins involved are poorly conserved across species. A physiologically relevant animal model would need not only to recapitulate the dyslipidemia of human familial hypercholesterolaemia, but also produce the regulatory proteins and key components of human lipid metabolism that do not otherwise exist in mice, such as cholesteryl ester transfer protein (CETP) and apolipoprotein (a) (APO(a)). Variants of the APO(a) gene are strongly associated with coronary artery disease, making it an important therapeutic target^10^. CETP is the single most robust genetic determinant of HDL levels in humans^11^. CETP mediates the exchange of cholesterol esters in HDL for triglycerides in very-low-density lipoprotein (VLDL) and intermediate-density lipoprotein (IDL): high levels of CETP activity result in a net movement of cholesterol esters from HDL to LDL, thereby lowering HDL cholesterol. CETP activity in plasma varies across different species: rabbits, hamsters and monkeys have CETP activity, but mice and rats lack the gene^12^,^13^. Since human hepatocytes synthesize and secrete these and many other key proteins of lipoprotein metabolism, xenografting human hepatocytes from familial hypercholesterolaemia patients into mice can potentially circumvent the disparity between the species.

The FRG (*fah*^−/−^*, rag2*^−/−^ and *Ilr2g*^−/−^) mouse was recently developed^14^,^15^. The severely immunodeficient mouse strain (T-, B- and NK-cell deficient) fosters repopulation with human hepatocytes by induction of cell autonomous hepatotoxicity in fumaryl acetoacetate hydrolase-deficient (*fah*^−/−^) mouse hepatocytes. In the absence of fumaryl acetoacetate hydrolase (FAH) activity, murine hepatocytes accumulate toxic tyrosine catabolites, while the human cells are protected through the FAH ortholog. The drug NTBC^16^ can regulate this selection pressure against murine hepatocytes by blocking tyrosine catabolism further upstream (hydroxyphenylpyruvate dioxigenase inhibitor) and leads to accumulation of nontoxic excretable metabolites. We have shown that human chimerism as high as 95% can be achieved in the FRG mouse^17^.

Here we demonstrate the successful establishment of a metabolic xenograft model for familial hypercholesterolaemia by transplanting human familial hypercholesterolaemia hepatocytes into FRG mice. The hypercholesterolaemia was rescued on experimental gene therapy and demonstrates the power of this novel platform for disease modeling.

## Results

### Establishing a familial hypercholesterolaemia xenograft model

**Figure 1:**
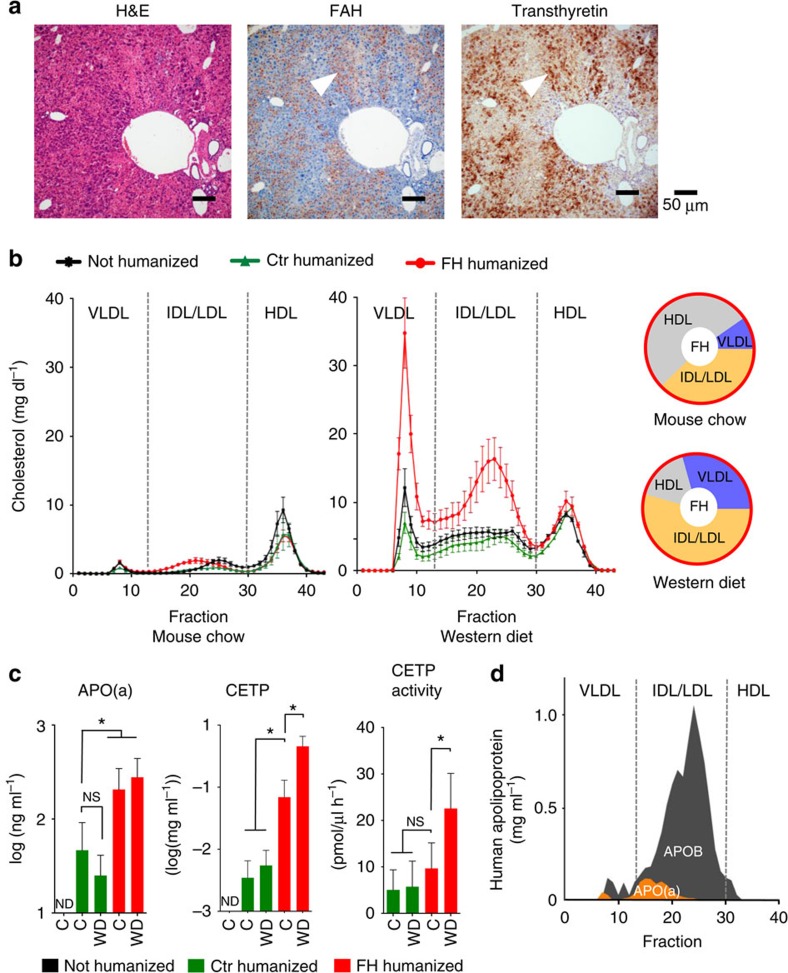
Hepatocytes from human familial hypercholesterolaemia (FH) induce hypercholesterolaemia in mice. (**a**), Serial sections of chimeric mouse liver repopulated with human FH hepatocytes showing hematoxylin and eosin staining (left panel), immunostaining for fumaryl acetoacetate hydrolase (FAH) (FRG mouse is *fah*^−/−^) (middle), and human-specific transthyretin (right). Human hepatocytes are positive for both markers (arrowheads). (**b**), Lipoprotein profiles of non-humanized FRG mice (*n*=3), Ctr chimeric mice (repopulated with normal human hepatocytes; green; *n*=3), and FH chimeric mice (repopulated with human FH hepatocytes; red; *n*=3) on normal mouse chow, which is low in cholesterol, or Western Diet (WD), which mimics the cholesterol-rich diet of humans. Pie charts show the distribution of cholesterol in FH chimeric mice on normal mouse diet and WD. (**c**), The plasma of FH and Ctr chimeric mice showed robust expression of human-specific apolipoprotein a (APO(a)), cholesteryl ester transfer protein (CETP), and CETP activity on Western Diet (WD) and mouse chow (C) (**d**), APO(a) is covalently bound to APOB and co-eluted in size-exclusion chromatography of FH chimeric plasma. Graph bars represent mean±standard deviation; * indicates *P*<0.05 by ANOVA resp. Mann–Whitney test; n.s., not significant; n.d., not detectable.

We isolated human hepatocytes from a 7-year-old familial hypercholesterolaemia patient who underwent liver transplantation. The patient's total serum cholesterol levels before transplantation were 800–1,000 mg dl^−1^; she had already developed cardiovascular disease despite lipid-lowering drug therapy (Supplementary Table 1), and genetic testing revealed compound heterozygosity (Cys134Tyr and Cys368Tyr) in the *LDLR* gene (Supplementary Table 2) and an apolipoprotein E (APOE) genotype E3/E3. Her hepatocytes were isolated from the explanted liver (viability 90%, trypan blue exclusion) and cryopreserved or directly transplanted via splenic injection into the liver of 1- to 2-month-old FRG mice as described previously^17^. As a control, we transplanted another group of FRG mice with commercial cadaveric human hepatocytes. We assessed the degree of repopulation in FRG mice transplanted with either human familial hypercholesterolaemia hepatocytes (hereafter referred to as familial hypercholesterolaemia chimeric mice) or normal human hepatocytes (hereafter referred to as Ctr chimeric mice) by measuring secreted human albumin in the murine serum^17^ and by immunostaining for the human-specific markers—FAH and transthyretin—in the liver (Fig.1a). As shown previously for normal human hepatocytes^17^, cryopreserved familial hypercholesterolaemia hepatocytes could also be used as a source for generating familial hypercholesterolaemia chimeric mice.

We then examined plasma lipoprotein profiles using size-exclusion chromatography from non-chimeric, Ctr and familial hypercholesterolaemia chimeric animals. Only chimeric mice with high human chimerism (>70% humanization) were used (Supplementary Fig. 1). Measurements revealed only minor differences between non-chimeric, familial hypercholesterolaemia and Ctr chimeric mice on normal mouse chow (Fig. 1b). The lipoprotein profiles of all three groups followed a predominantly murine pattern, with most of the cholesterol being in the high-density (HDL) fraction. Since standard mouse chow is very low in cholesterol, 6 to 8 months after transplantation we switched all three groups of mice to Western diet, a commonly used cholesterol-rich high-fat diet that mimics the dietary intake of humans in industrialized Western nations. Within 4 weeks of starting Western diet, cholesterol accumulated in the very low density (VLDL) and intermediate density (IDL)/LDL fractions of all groups. Only familial hypercholesterolaemia chimeric mice, however, developed pathological levels of cholesterol (total cholesterol: 321–605 mg dl^−1^) due to a significant increase in the non-HDL fractions (Fig. 1b). The non-HDL cholesterol fraction in familial hypercholesterolaemia chimeric mice increased from 47% on mouse chow to 84% on Western diet. After the first month, cholesterol levels in the familial hypercholesterolaemia chimeric mice stabilized and did not increase further with maintenance on Western diet (Supplementary Fig 2). The major human apolipoproteins (A-I (APOA), B-100 (APOB) and APOE) could be readily detected in the murine serum (Supplementary Fig. 3). We were also able to detect other human apolipoproteins such as apolipoprotein A-II, D or C-III when performing mass spectrometry on HDL and LDL fractions (Supplementary Table 3).

### Identifying conserved elements of human lipoprotein metabolism

We then turned to detection of key proteins for human lipid metabolism in humanized mice. APO(a) is found only in old-world monkeys and humans^18^,^19^. APO(a) is covalently bound to APOB on a lipoprotein subclass referred to as Lipoprotein (a) (Lp(a)). High plasma levels of Lp(a) are directly associated with increased risk of cardiovascular disease^10^,^20^. We readily detected APO(a) in the plasma of both familial hypercholesterolaemia and Ctr chimeric mice (Fig. 1c); familial hypercholesterolaemia chimeric mice showed higher levels of APO(a), which could be due to the absence of LDLR or to isoform differences between donor hepatocytes in this highly polymorphic protein. Protein analysis of plasma fractions from chimeric mice revealed co-elution compatible with association of APO(a) and APOB, confirming observations in humans (Fig. 1d).

CETP is a key protein in human reverse cholesterol transport and an emerging drug target. We detected CETP protein in the plasma of all human liver chimeric mice, with highest levels in familial hypercholesterolaemia chimeric mice fed Western diet. CETP activity closely mirrored CETP protein levels, demonstrating its functionality in the murine circulation (Fig. 1c). Furthermore, CETP activity in serum of familial hypercholesterolaemia chimeric mice fed Western diet was comparable to that observed in human control serum (range: 23.4–28.6 pmol μl^−1^ h^−1^).

### Gene therapy with adeno-associated virus (AAV)

**Figure 2:**
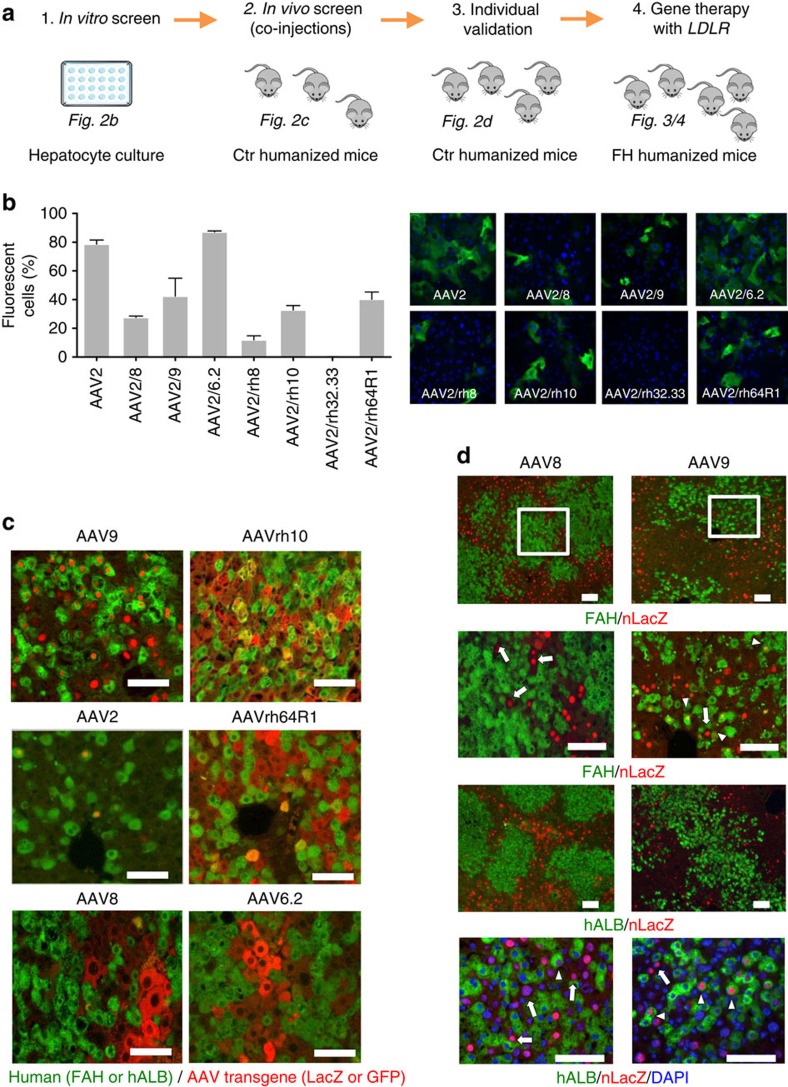
AAV9 shows superior transduction of human hepatocytes. (**a**), Strategy for AAV serotype transduction analysis. (**b**), *In vitro* comparison of the transduction efficiency of different AAV serotypes in primary human hepatocytes. AAV vectors expressing EGFP driven by the liver-specific promoter TBG (AAV.TBG.EGFP) were inoculated with primary human hepatocytes at an MOI of 10^6^ GC per cell for 4 days, after which cells were fixed and micrographs taken under direct fluorescence at identical exposure settings for all vectors. The bar graph shows quantitative morphometric analysis of the transduction efficiency of each AAV based on per cent transduction of hepatocytes; bars represent mean±s.d., *n*=3 for each serotype. (**c**), Representative liver sections of chimeric mice repopulated with normal human (Ctr) hepatocytes (in green, ALB or FAH immunostaining), that were co-transduced by intravenous injection (3x10^11^ GC per mouse) of two different AAV serotypes expressing either GFP or LacZ (in red, nuclear targeted LacZ for AAV9 and AAV2 or cytoplasmic LacZ for AAV8). (**d**), Comparison AAV9 to AAV8 in transduction efficiency for human hepatocytes in chimeric FRG mice. Chimeric mouse liver transduced with AAV9 or AAV8 CB.*nLacZ*, co-stained for the AAV derived transgene LacZ in red and either human albumin (ALB) or fumaryl acetoacetate hydrolase (FAH) in green. The insets (white squares) in the upper panels are magnified in the lower panels. The two lowest panels show co-staining of nuclear DAPI and lacZ of human and mouse cells. Arrows point to transduced mouse hepatocytes and the arrowheads at human hepatocytes. Scale bar is 50 μm.

We sought to restore healthy lipid metabolism in familial hypercholesterolaemia chimeric mice by applying human gene therapy. Recombinant AAV is a promising vector for safe and long-term gene expression in non-dividing cells such as hepatocytes, myocytes or neurons^21^. Since the tropism of AAV is serotype (capsid) dependent^22^, we first validated previously isolated AAV capsids from nonhuman primates and human tissues^23^,^24^,^25^, a subset of which has been evaluated in mouse, dog, and nonhuman primates for liver-directed gene transfer^26^,^27^,^28^, for transduction of human hepatocytes (Fig. 2a). To narrow the candidate AAV serotypes for *in vivo* validation, we transduced primary human hepatocytes in tissue culture (Fig. 2b) and then injected the six AAV serotypes with the highest transduction efficiencies into the tail veins of Ctr-chimeric mice: two different AAV serotypes (3 × 10^11^ GC per mouse for each vector), each containing either a *GFP* or *LacZ* reporter, into each of three humanized mice. Ten days after injection we collected the animals and immunostained chimeric livers for human hepatocyte markers (FAH or human ALB) and the transgene LacZ or GFP (Fig. 2c). AAV9 and AAVrh10 were the most efficient at transducing human hepatocytes *in vivo*. Since we had to use different expression cassettes in this co-injection screen, we decided to further evaluate the effect of the promoter and reporter for AAV9 and the current clinical standard, AAV8, using an identical expression cassette (AAV-CB.*nLacZ*). This time, we injected only one AAV per chimeric mouse. Again, AAV9 reveals the highest transduction efficiency (Fig. 2d), confirming the data from the co-injection experiment. AAV9 transduction of human hepatocytes was not promoter or reporter dependent (Supplementary Fig. 4).

### AAV9–LDLR rescues hypercholesterolaemia

**Figure 3:**
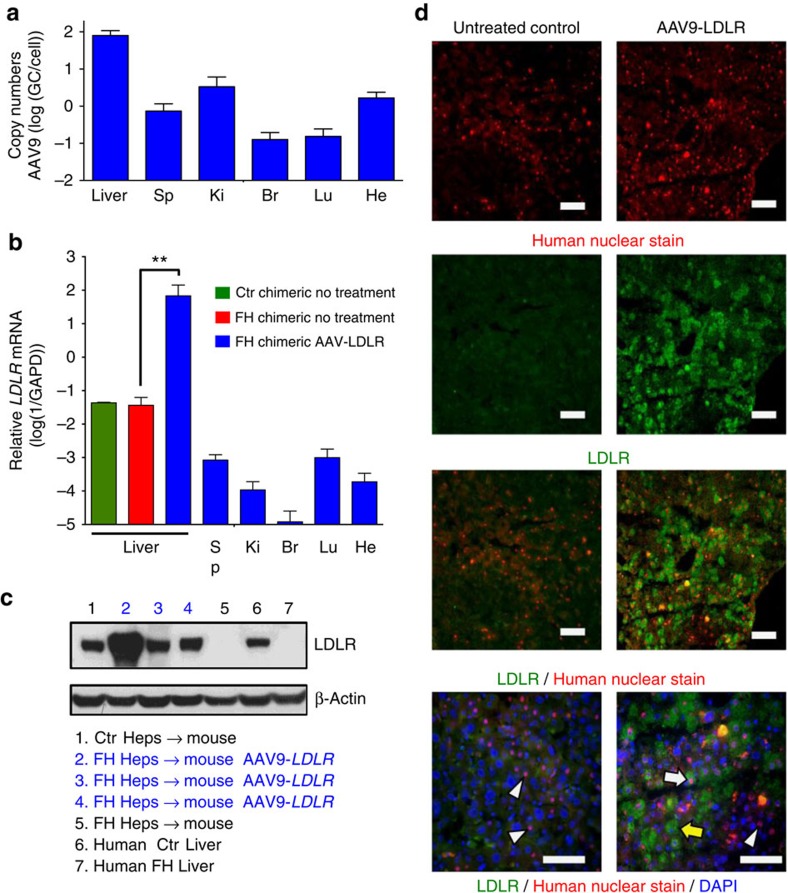
AAV9–LDLR rescues defective LDLR in FH liver chimeric mice. (**a**), Biodistribution of AAV9–LDLR in different organs. qPCR of liver, spleen, kidney, brain, lung and heart of three familial hypercholesterolemia (FH) chimeric mice 30 days after transduction with AAV9–*LDLR*. Mean copy numbers±s.d. are shown. (**b**), qRT–PCR shows expression of human *LDLR* in the liver but not extrahepatic organs in FH chimeric mice transduced with AAV9.TBG.*hLDLR*. (**c**), Western blot reveals human LDLR in treated FH chimeric mice and control groups, demonstrating rescue of genetic defect underlying FH. (**d**). Immunostaining of FH chimeric livers for LDLR (green) and human nuclei (red). Only AAV9–LDLR treated mice show staining for LDLR in human (white arrow) and in mouse (yellow arrow) hepatocytes, while LDLR negative human cells (arrowhead) can be found in both treated and untreated FH chimeric mice. Br, brain; He, heart; Ki, kidney; Lu, lung; Sp, spleen. Scale bar is 50 μm, ** indicates *P*<0.01 by *t*-test.

On the basis of AAV serotype analysis, we then produced AAV9 vectors bearing the human *LDLR* gene under a strong liver-specific promoter (thyroxine-binding globulin, TBG), and injected them (3 × 10^11^ GC per mouse) into the tail veins of familial hypercholesterolaemia chimeric mice (*n*=5). Thirty days after injection, biodistribution of the gene therapy vector was measured in the liver and some extrahepatic organs (kidney, spleen, heart, lung and brain) and, as expected, the liver contained the most vector genomes (47–97 GC per diploid genome) supporting the liver tropism of AAV9 (Fig. 3a). These results are consistent with the vector dose used and previously reported data on AAV9 vector distribution in mouse tissues following systemic delivery^29^. Transcriptional analysis of chimeric livers and extrahepatic organs revealed substantial overexpression of human *LDLR* mRNA in the liver only (Fig. 3b, Supplementary Fig. 5). Furthermore, the treated mice exhibited robust expression of LDLR protein, which was not detectable in untreated familial hypercholesterolaemia chimeric mouse livers, the human familial hypercholesterolaemia donor liver (Fig. 2c, Supplementary Fig. 6) or in extrahepatic organs from treated familial hypercholesterolaemia chimeric mice (Supplementary Fig. 5). Most importantly, LDLR could be detected in 15–25% of human and 25–35% mouse hepatocytes in AAV9–*LDLR* treated familial hypercholesterolaemia chimeric animals (Fig. 2d). Although AAV9 transduced more efficiently mouse cells, in analysed sections the majority of transduced hepatocytes (50–95% depending on local degree of humanization) were human hepatocytes.

**Figure 4:**
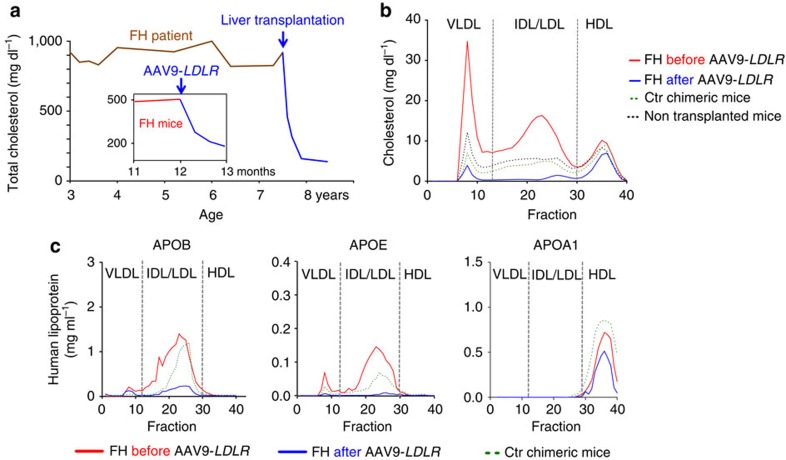
Gene therapy rescues lipid profiles of FH chimeric mice. (**a**), Treated FH chimeric mice (inset; *n*=5) experienced reduction of total cholesterol levels comparable to that observed in FH hepatocyte donor after transplant. (**b**), Mean cholesterol levels in serum fractions before (red, *n*=3) and 4 weeks after (blue, *n*=3) injection of AAV9–*LDLR* in FH chimeric mice. Non-HDL cholesterol levels were reduced to below those of both Ctr chimeric mice (dotted green line) and non-humanized mice (dotted black line). (**c**), Mean human APOB, APOE and APOA1 levels before (red) and 4 weeks after therapy (blue) in plasma fractions (*n*=3, each group). Levels of APOA1 were similar before and after therapy, while APOB and APOE were significantly reduced by AAV–LDLR.

Total plasma cholesterol levels decreased within 4 weeks after AAV9–*LDLR* injection from 509±58 (mean±s.d.) to 179±13 mg dl^−1^ (*P*<0.001). Figure 4a places the response of familial hypercholesterolaemia chimeric mice to gene therapy in the context of the response of the familial hypercholesterolaemia hepatocyte donor to orthotopic liver transplantation: both treatments restored normal total serum cholesterol levels, with the gene therapy normalizing levels over the course of 1 month. Blood samples from familial hypercholesterolaemia chimeric mice (pretreatment and 4 weeks post therapy, *n*=3), Ctr (*n*=3) and non-humanized mice (*n*=3) were subjected to size-exclusion chromatography. Cholesterol levels across different fractions demonstrated that all treated familial hypercholesterolaemia chimeric mice experienced a significant reduction (*P*<0.01) in non-HDL cholesterol (Fig. 4b). In fact, after AAV9–*LDLR* treatment, familial hypercholesterolaemia chimeric mice had significantly (*P*<0.05) lower cholesterol levels than non-treated Ctr chimeric and non-humanized mice, indicating that the treated liver is able to remove lipoprotein particles from the blood.

LDLR reduces serum cholesterol levels through the uptake of lipoprotein particles mediated by the high (APOE) and low (APOB) affinity ligands. We therefore measured the two ligands before and after AAV9–*LDLR* gene therapy. AAV9–*LDLR* treatment removed 84% of serum APOB levels, primarily from the IDL/LDL fraction, and APOE levels were decreased even more, by 95% (Fig. 4c). We also measured apolipoprotein A1 (APOA1), which is the main structural component of HDL particles and not a ligand for LDLR, and, as expected, AAV9–*LDLR* treatment did not significantly alter its plasma levels (Fig. 4c).

## Discussion

Since their first description^30^,^31^, human liver chimeric mice have been used to study drug metabolism^32^,^33^, viral hepatitis^17^,^30^,^31^ and regenerative medicine^34^,^35^. The present work extends this list to metabolic disorders by establishing a human xenograft model for familial hypercholesterolaemia. Although it has been shown that human hepatocytes from metabolic disease patients can be transplanted into immune-deficient mice^36^, induction of a human disease phenotype upon hepatocyte transplantation had not been achieved. Here we show that familial hypercholesterolaemia chimeric mice demonstrate a ‘humanized' lipoprotein profile with a hypercholesterolaemia similar to that observed in human familial hypercholesterolaemia patients. Moreover, key regulators and emerging drug targets such as CEPT and APO(a) could be detected and quantified in chimeric mice, and the dyslipidemia was corrected with AAV9–*LDLR* gene therapy. Familial hypercholesterolaemia chimeric mice thus provide a promising platform to evaluate experimental therapies for patients. Homozygous, or compound heterozygous familial hypercholesterolaemia patients like our patient are unresponsive to statin therapy and alternatives to apheresis or liver transplantation are desperately needed. The absence of LDLR protein in the familial hypercholesterolaemia donor liver and chimeric mice liver suggests these alleles may be class 1 mutations^37^ which are receptor negative—possibly due to unstable protein. While mutations of both cysteine residues are associated with familial hypercholesterolaemia in humans (Supplementary Table 2), biochemical characterization of these variants is outside the scope of this work.

Although familial hypercholesterolaemia chimeric mice showed pathological lipid profiles in contrast to Ctr chimeric mice, hypercholesterolaemia in familial hypercholesterolaemia patients is generally higher, as was also the case for our donor before liver transplantation (Fig. 4a). Possible explanations for this discrepancy are (1) extrahepatic clearance of LDL by mouse Ldlr in peripheral tissues^38^, and (2) the Ldlr activity of residual mouse hepatocytes present in the chimeric livers. The complex interaction of the host and transplanted hepatocytes might limit the utility of the xenograft model for metabolic diseases involving soluble, membrane-permeable metabolites.

We conducted a survey of various AAV vector expressing reporter genes in animals repopulated with normal human hepatocytes (Ctr chimeric mice). This model was recently used to identify variant AAV vectors with high liver tropism^39^. In the present work, vectors demonstrated varying levels of human and endogenous mouse hepatocyte transduction, although in each case transduction was more efficient in mouse hepatocytes than in human hepatocytes. This might explain why previous studies in Ldlr knockout mice required lower vector doses to normalize serum cholesterol levels^40^,^41^. For LDLR gene transfer in this model we proceeded with a liver-specific TBG promoter and an AAV9 capsid, which yielded expression in human hepatocytes when tested four times in three different configurations. One injection of AAV9–LDLR led to normalization of total cholesterol levels within 4 weeks, and non-HDL cholesterol levels were reduced to below those of both Ctr chimeric mice and non-humanized mice (Fig. 4). This remarkable rescue of hypercholesterolaemia was achieved with only 15–35% of hepatocytes (mostly human) expressing the LDLR. These data raise the question of whether or not AAV9–LDLR gene therapy is a valuable alternative to orthotopic liver transplantation in homozygous familial hypercholesterolaemia patient. To estimate the clinical benefit of present gene therapy approach, other parameters like long-term gene expression and immunological compatibility are needed, but neither can be validated in the familial hypercholesterolaemia xenograft model due to the limited experimental window and the lack of a functional immune system. The familial hypercholesterolaemia xenograft model cannot replace clinical studies, nor can it give a definitive answer about the clinical utility of a new therapy. Nevertheless, this preclinical model is a powerful new tool to translate experimental therapies more efficiently and get a first validation in a ‘human setting'.

This proof-of-principle study demonstrates the feasibility of modelling and treating a human metabolic disease in mice. We envision the use of xenograft models for the study of many metabolic diseases, including urea cycle defects and lysosomal storage disorders. Induction of human disease in mice is dependent on the residual activity of the affected enzyme/receptor, alternative intra- and extrahepatic pathways and ‘cross-talk' between graft (human) and host (mouse). Achieving a high human chimerism is another prerequisite for a metabolic xenograft model. Not all metabolic disease hepatocytes are likely suitable for repopulation. Metabolic stress, such as Western diet, might interfere with reaching efficient repopulation rates. Last, metabolic xenograft models are unlikely to recapitulate every aspect of human disease. For instance, familial hypercholesterolaemia chimeric mice did not develop arteriosclerotic alterations, likely because of their impaired immunity^42^ as well as their short time period on Western diet (<4 months). Nevertheless, the familial hypercholesterolaemia chimeric mice recapitulate the majority of human lipoprotein metabolism and should be ideally suited to evaluating novel cholesterol-lowering therapies. With a single AAV9–LDLR dose we could normalize serum cholesterol levels and thereby demonstrate the power of this new xenograft platform for metabolic diseases.

## Methods

### Human hepatocyte isolation and transplantation into FRG mice

We isolated human familial hypercholesterolaemia hepatocytes by the two-step collagenase perfusion method of Berry and Friend^43^ with the modification of Segelen 44. In brief, we cannulated the largest portal veins with a silicon tubing system connected to a peristaltic pump, then flushed the liver with ice-cold basic perfusion solution (BPS: 10 mM Hepes buffer) followed by perfusion with BPS containing 0.5 mM EGTA to prevent the formation of blood clots. The liver was then perfused with warm collagenase solutions (2 mg dl^−1^ collagenase) until the organ became soft. We cut the liver into small pieces (2–3 cm^3^) and released hepatocytes into the solution by applying minor shear stress (with forceps) on the pieces. Hepatocytes were immediately washed in ice-cold BPS containing 0.5% BSA and centrifuged (three times 50 × *g*, 5 min). Viability was assessed by trypan blue exclusion. Normal human hepatocytes for Ctr chimeric mice were purchased from Triangle Research Labs, NC.

Hepatocytes (3 × 10^6^ per mouse) were transplanted into the murine liver of FRG mice by splenic injections as originally described for mouse hepatocytes^45^. In brief, the abdominal cavity was opened by a midabdominal incision, and 3 × 10^6^ human hepatocytes in a volume of 100 μl PBS were injected into the spleen. Immediately after transplantation, selection pressure towards transplanted human hepatocytes was applied by withdrawing the drug NTBC from the drinking water in the following steps: 2 days at 25%; then 2 days at 12% and eventually 2 days at 6% of the colony maintenance dose (100%=7.5 mg l^−1^) before discontinuing the drug completely^17^. To determine the extent of human chimerism, we measured human albumin (ELISA, Bethyl laboratories) in the murine blood, having previously shown that human albumin levels correlate with the level of human chimerism assessed by immunostaining of human hepatocytes^17^. Only mice with a human chimerism >70% were used for AAV injections. Some human liver chimeric mice were put where indicated on Western diet: D12079B from Research Diets. The IRB and IACUC of Baylor College of Medicine approved procurement of human familial hypercholesterolaemia liver and animal experiments, respectively. Informed consent was obtained from the familial hypercholesterolaemia patient.

### AAV vector production and injection into chimeric FRG mice

Recombinant AAV vectors expressing GFP or β-galactosidase (LacZ, nuclear targeted or cytoplasmic) were driven by CMV-enhanced chicken β-actin or TBG promoter and packaged with one of eight capsids (AAV2, 6.2, 8, 9, rh8, rh10, rh32.33, and rh64R1) for comparison of efficacy. AAV9 was used to package human *LDLR* driven by TBG promoter (AAV.TBG.*hLDLR*). Viral production and purification were performed by the Penn Vector Core at the University of Pennsylvania as described previously^46^. In brief, 75% confluent monolayer of HEK293 cells in 10-layer cell stacks were transfected by polyethylenimine (PEI)-based triple transfections with three plasmids at a ratio of 2:1:1 (1092 μg of adenovirus helper plasmid/546 μg of cis plasmid/546 μg of trans plasmid per cell stack). The PEI /DNA ratio was maintained at 2:1 (w/w) and added to serum-free DMEM. Vectors were first concentrated from culture medium 5 days after transfection by tangential flow filtration and further purified by iodixanol gradient ultracentrifugation. The genome titre (GC per ml) of AAV vectors were determined by real-time PCR (rt-PCR) using a primer/probe set corresponding to the bovine growth hormone polyA region of the vector and linearized plasmid standards (For primer 5′-GCCAGCCATCTGTTGT-3′, Rev primer 5′-GGAGTGGCACCTTCCA-3′, probe 5′-6FAM—TCCCCC GTGCCTTCCTTGACC—TAMRA-3′). All vectors used in this study passed the endotoxin assay using QCL-1000 Chromogenic LAL test kit (Cambrex Bio Science, Walkersville, MD). Vectors were injected into the tail veins of chimeric (familial hypercholesterolaemia and Ctr humanized) mice with 100 μl PBS containing 3 × 10^11^ GC.

### Size-exclusion chromatography

Plasma lipid profiling was performed by the Mouse Metabolic Core (MMC) at BCM. Plasma collected from individual mice was fractionated by FPLC using two Superose-6 columns connected in series (Pharmacia FPLC System, Amersham Pharmacia Biotech). Fractions of 0.5 ml were collected in an elution buffer as we have previously described^47^.

### Plasma measurements

Human APOB (Mabtech Inc. 3715-1HP-2), APOA-1(Mabtech Inc., 3710-1HP-2), APOE (Mabtech Inc. 3712-1HP-2), APO(a) (Cell Biolabs, STA-359), CETP (Alpco Diagnostics, 47-CETHU-E01) and ALB (Bethyl Laboratories, E88-129) were measured on whole plasma or lipoprotein fractions according to the manufacturer's recommendation. CETP activity (Biovision, K601-100) was measured with 2 μl of plasma. Cholesterol of lipid fractions and whole blood was measured with a colorimetric method (Cholesterol E, Wako Diagnostics, 439–17,501).

ELISA was performed of three or more biological replicates (mean of experimental duplicates) per group. CETP activity was determined from three biological replicates, each having three experimental replicates. Cholesterol of each fraction was single measurements of three biological replicates per group.

### Immunostaining chimeric livers

Immunostaining was performed on three different types of samples: formalin-fixed paraffin-embedded liver (FAH, FAH and GFP, FAH and LacZ and human transthyretin), unfixed-frozen liver (nuclear targeted LacZ, human ALB, human nuclear staining and LDLR) and fixed-frozen liver (human ALB and GFP). Each of these methods proved to give the most sensitive results for the detection of the respective reporter or human protein.

Paraffin sections were dewaxed and antigen retrieval was performed in citrate buffer pH6.0. Sections from unfixed liver frozen in OCT compound were fixed in 4% paraformaldehyde/PBS. Fixed-frozen liver was prepared after overnight fixation in formalin, washing in PBS and freezing in OCT compound before sectioning. Immunostaining was performed after blocking with 1% donkey serum+0.2% Triton with rabbit antibodies against GFP (Fitzgerald Industries, 20R-GR011, diluted 1:500) or LDLR^48^ (1:200), goat antibodies against FAH (Santa Cruz, sc-66223, diluted 1:50), chicken antibodies against LacZ (Abcam ab9361, diluted 1:200), goat antibodies against human ALB (Bethyl Laboratories, A80-129A, diluted 1:200), mouse antibodies against human nuclei (Millipore, MAB1281, diluted 1:200) and rabbit antibodies against transthyretin (Dako, A0002, diluted 1:500). After washing in PBS, the sections were stained with fluorescent-labelled secondary antibodies (Jackson Immunoresearch Laboratories, Alexa594 goat anti mouse 115-585-003, diluted 1:1,000; Alexa488 goat anti-rabbit 111-545-144, diluted 1:1,000; Cy5 donkey anti-goat 705-025-147 diluted 1:200; FITC donkey anti-rabbit 711-095-152 diluted 1:100; TRITC donkey anti-chicken 703-025-155, diluted 1:100) in 1% donkey serum for 30 min, washed again and mounted with Vectashield plus DAPI (Vector Labs). Human transthyretin and some FAH detections were done according to the manufacturer's recommendations of the Vectastain ABC kit (Vector Labs), followed by the DAB detection kit (Vector Labs) and counterstaining with hematoxylin.

### Quantification transduction efficiencies AAV serotypes

Quantification of transduction was performed in two ways. For livers expressing nuclear targeted LacZ, 11 sets of images (showing each LacZ, human ALB and nuclear DAPI staining) per animal and vector were taken with a 10 × objective. The percentages of transduced human and mouse hepatocytes were calculated for every image set by combining the following individual measurements:

(1) The number of LacZ positive human hepatocytes was established by direct counting on overlay pictures, whereas the number of LacZ positive mouse cells was established by first determining the total number of LacZ positive cells per image with the help of ImageJ software (W. Rasband, National Institutes of Health, Bethesda, MD; http://rsb.info.nih.gov/ij) and then subtracting the established value for positive human cells.

(2) The percentage of area occupied by human hepatocytes (human ALB positive) and mouse hepatocytes (human albumin negative) was determined through thresholding and area measurement with ImageJ. Weak background fluorescence allowed human ALB-negative cells to be distinguished from cell-free areas such as veins and sinusoids.

To establish the correlation between area percentage value and number of hepatocytes, DAPI stained nuclei of hepatocytes were counted with ImageJ (a minimum pixel number of 50 was used to ignore small nuclei from other cells) for mouse and human cells, respectively. For human hepatocytes a value of 2,708 hepatocytes/100% image area and for mouse hepatocytes a value of 1,789 hepatocytes/100% image area was determined, reflecting a smaller size of the human cells in the FRG model. Finally, the percentages of transduced human and mouse hepatocytes were calculated for every image from the above measurements and the average for each animal and vector was determined.

For livers expressing cytoplasmic LacZ or GFP, 5–9 images from liver sections stained for LacZ and FAH, LacZ and human ALB, GFP and FAH, and GFP and human ALB were quantified by area. The percentage of image area positive for each protein was established after thresholding with ImageJ. Next, the thresholded images showing the expression of the reporter gene were combined with the corresponding thresholded images showing human hepatocytes (corresponding to FAH- or human ALB-positive area) or mouse hepatocytes (corresponding to FAH- or human ALB-negative area not including cell-free areas such as blood vessels). This was done with ImageJ's image Calculator by image addition of tresholded images where the thresholded pixels equal 0 and the remaining pixels equal 255 (so that 0+0=0, that is, only the overlap area between two images remains the value 0 in the resulting image). The overlap area (that is, pixels with value 0 showing LacZ or GFP-positive human or mouse cells) was finally quantified and the percentage of GFP or LacZ-positive human and mouse cells was established.

### LDLR western blotting and qRT–PCR

Fresh frozen liver samples were thawed, cut into small pieces and treated with a homogenizer in standard lysis buffer. Then 20 μg protein were subjected to SDS–PAGE (4–12% Bis-Tris Gel (Invitrogen)) followed by transfer to a PVDF membrane. The membrane was blocked in 5% milk and immunostained for LDLR using a rabbit anti-LDLR antibody^48^ and mouse anti-β-actin (SIGMA). Total RNA was isolated using Purelink RNA mini kit (Ambion). cDNA was reverse transcribed from 4 μg of total RNA using qScript cDNA Supermix (Quanta Biosciences). rt-PCR reactions were performed using iTaq Universal SYBR Green Supermix (Bio-Rad) and analysed on an ABI Prism 7900HT Sequence Detection System (Applied Biosystems). All data was normalized to GAPDH as an internal control, in the chimeric liver to human GAPDH and in extrahepatic organs to mouse GAPDH. Two published *LDLR* primer sets were used: 5′-GCTTGTCTGTCACCTGCAAA-3′ and 5′-AACTGCCGAGAGATGCACTT-3′^49^ and, 5′-GCGTGAAATTGCGCTGGACCGTC and 5′-TCACAGACGAACTGCCGAGAGATGC-3′^50^.

### Vector biodistribution analysis

Tissue DNAs were extracted using QIAamp DNA Mini Kit (Qiagen, Valencia, CA). Detection and quantification of vector genomes in extracted DNA were performed by rt-PCR as described previously using primers and probe set corresponding to the TBG promoter 51. For primer 5′-AAACTGCCAATTCCACTGCTG, Rev primer 5′-CCATAGGCAAAAGCACCAAGA-3′, probe 5′-6FAM—TTG GCCCAATAGTGAGAACTTTTT CCTGC—TAMRA-3′.

### Mass spectrometry analysis

The samples were precipitated with 80% acetone at −20 °C overnight twice to remove the lipids and salts. The protein pellets were resuspended in 0.2% ProteasMax surfactant (Promega Inc.) with 2 M urea (GE Health) in 50 mM ammonium bicarbonate pH 7.9. The protein quantity was measured using NanoOrange kit (Invitrogen Inc.) with BSA as protein standard. Trypsin (Sigma Inc.) were added to the samples in a ratio of protein:trypsin of 20:1. The samples were digested in 37 °C for 3 h. The peptide solutions were cleaned up using C18 clean-up spin column (Thermo Fisher Inc.). The eluents were dried and resuspended in 0.1% formic acid with 5% acetonitrile. The samples were quantified again using NanoOrange kit with BSA as protein standard. They were then normalized to 50 ng μl^−1^ and a total of 200 ng of total tryptic peptides were loaded to the Eksigent ultra2D nanoLC system with C18 cHiP system.

The Eksigent nanoLC system was running in buffer A (0.1% formic acid in water) and buffer B (0.1% formic acid in acetonitrile) as mobile phases. The samples were trapped and washed in the trap column chip for 10 min in 50% buffer A and 50% buffer B with a flow rate of 3 μl min^−1^. A 90 min gradient of 5% buffer B to 35% buffer B in 300 nl min^−1^ flow rate was used to separate peptides into different retention times. Buffer B was increased to 80% in 5 min and was maintained for 10 min before decreasing down to 5% in 5 min. The nanoLC system was then equilibrated with 95% buffer A and 5% buffer B for 20 min for the next sample.

The TripleTOF 5,600 mass spectrometer (ABSCIEX Inc.) was in line with Eksigent ultra2D system via the nanoflex spray under the control of Analyst TF 1.6 software (ABSCIEX Inc.). Q1 of the mass spectrometer was analysed from 400 to 1,250 amu. The curtain gas was at 2, spray voltage was set at 2,500 V and the spray temperature was set at 100 °C. The cycle was set at 0.25 s. Q3 was set at 100 to 2,000 amu and the cycle was set at 0.1 s. The raw WIFF files were converted to MGF files under Peakview v1.2 software (ABSCIEX Inc.). The MGF files were imported into PeakStudio v6 software (Bioinformatic Solutions Inc.) for protein identification, *de novo* sequencing, and post-translational modifications analyses. The search criteria were set as Mammalia in NCBI non-redundant database search to reveal mouse proteins versus human proteins. Identified proteins matching to non-mouse and non-human were filtered out.

### Statistics

All experiments were blinded, with following exceptions: Feeding, bleeding and collecting of mice, qRT–PCR, western blotting, AAV biodistribution in organs, AAV transduction efficiencies in tissue culture and LDLR immunostaining. Groups of two were analysed by *t*-test, or, when non-parametric, by the Mann–Whitney test. More than two groups were compared by ANOVA. Statistical analysis was performed using PRISM version 6.0 software (Graph Pad software). Statistical significance was assumed with *P* value <0.05 (*) and *P*<0.01(**). Bars in graphs represent s.d. for the group. Group size (*n*) represents biological samples and not experimental sample size. Sample size of familial hypercholesterolaemia chimeric mice was determined by transplantation capacity on the day of hepatocyte isolation and by achievement of high human chimerism (>70%). Familial hypercholesterolaemia and Ctr chimeric mice were allocated in groups by the degree of repopulation, so that each group had comparable degrees of human chimerism.

## Additional information

**How to cite this article:** Bissig-Choisat, B. *et al.* development and rescue of human familial hypercholesterolaemia in a xenograft mouse model. *Nat. Commun.* 6:7339 doi: 10.1038/ncomms8339 (2015).

## Supplementary Material

Supplementary Information Supplementary Figures 1-6, Supplementary Tables 1-3 and Supplementary References
