# Supplementary material for: Development and rescue of human familial hypercholesterolaemia in a xenograft mouse model
Source: Nat Commun. 2015 Jun 17;6:7339. doi: 10.1038/ncomms8339 (PMC4557302; doi:10.1038/ncomms8339)
Supplement: Supplementary Information — Supplementary Figures 1-6, Supplementary Tables 1-3 and Supplementary References [file ncomms8339-s1.pdf]

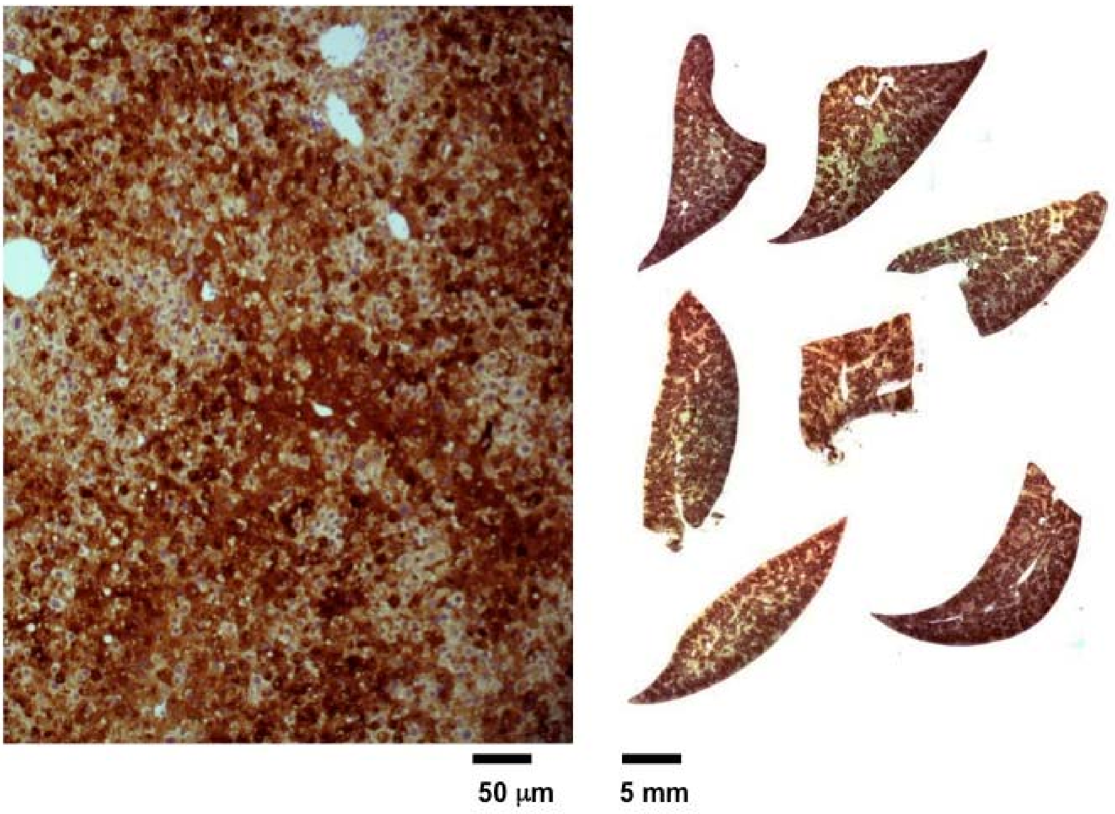

Supplementary Figure 1. High human chimerism in FRG mice repopulated with human FH hepatocytes. Representative immunostaining for human specific transthyretin shown in high (left) and low (right) magnification. The latter is a composed picture across multiple liver lobes of the same animal.

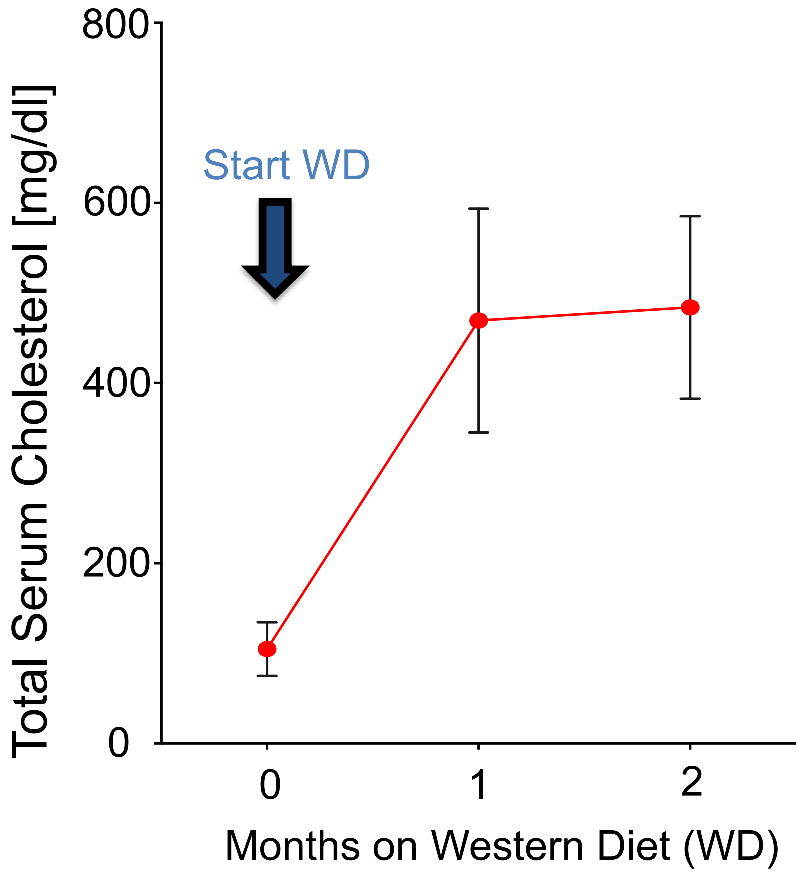

Supplementary Figure 2. Total Serum Cholesterol levels stabilize after 1 month on Western Diet (WD). FH chimeric mice (n=6) were put on Western Diet and bled for analysis of total serum cholesterol levels. Mean ±SEM are shown in graph.

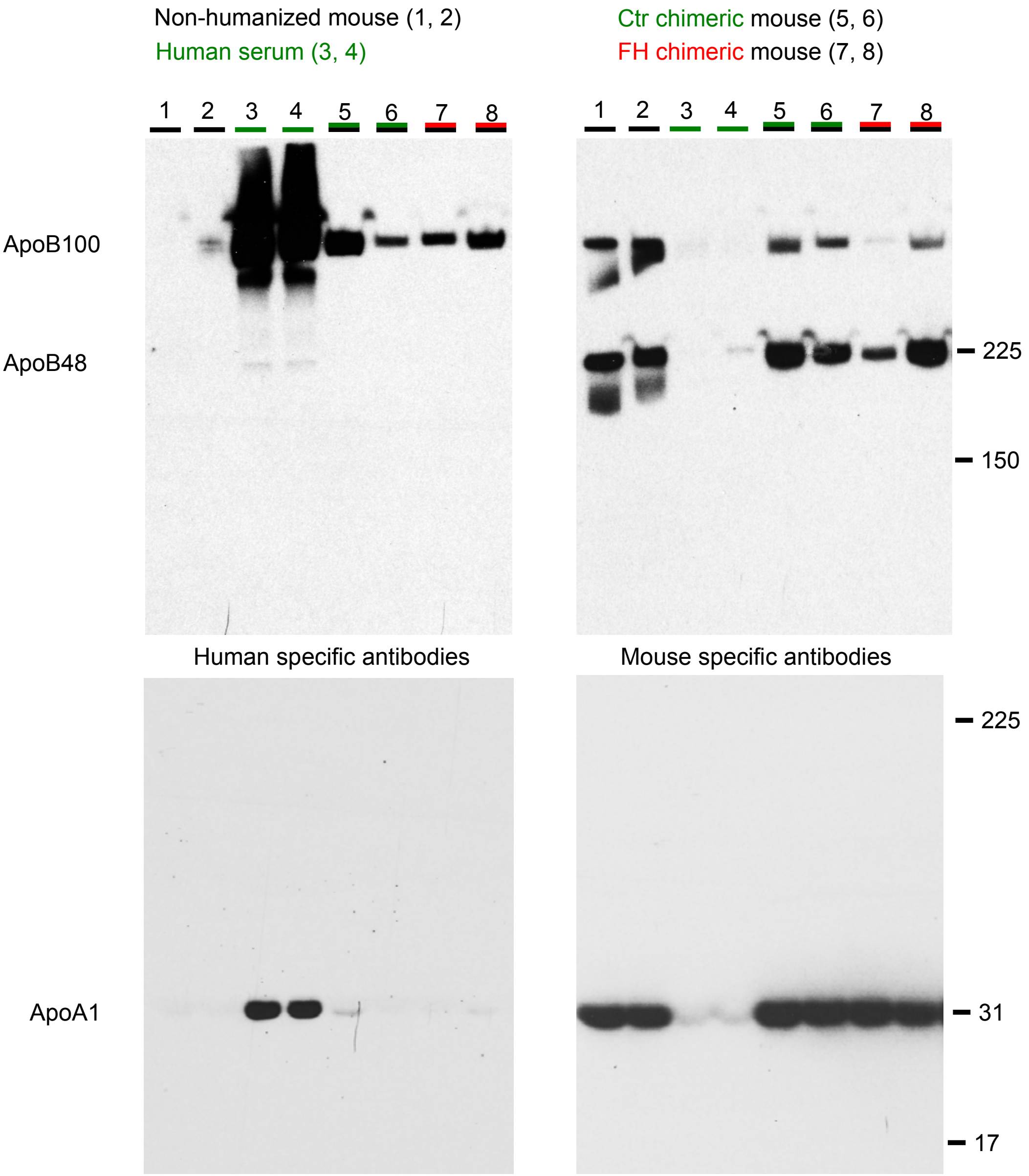

Supplementary Figure 3. Detection of mouse and human apolipoproteins in the plasma of human liver chimeric mice. Fasted plasma samples (as indicated) were used for Western blotting to detect human APOB (left panel) and murine ApoB (right panel). The same samples with identical amount of plasma were loaded for Western blotting using a human specific APOA1 (left) and a mouse specific ApoA1 (right) antibody. Note that human ApoB48 can not be detected in humanized mice and that APOA1 produced from human hepatocytes is difficult to detect.

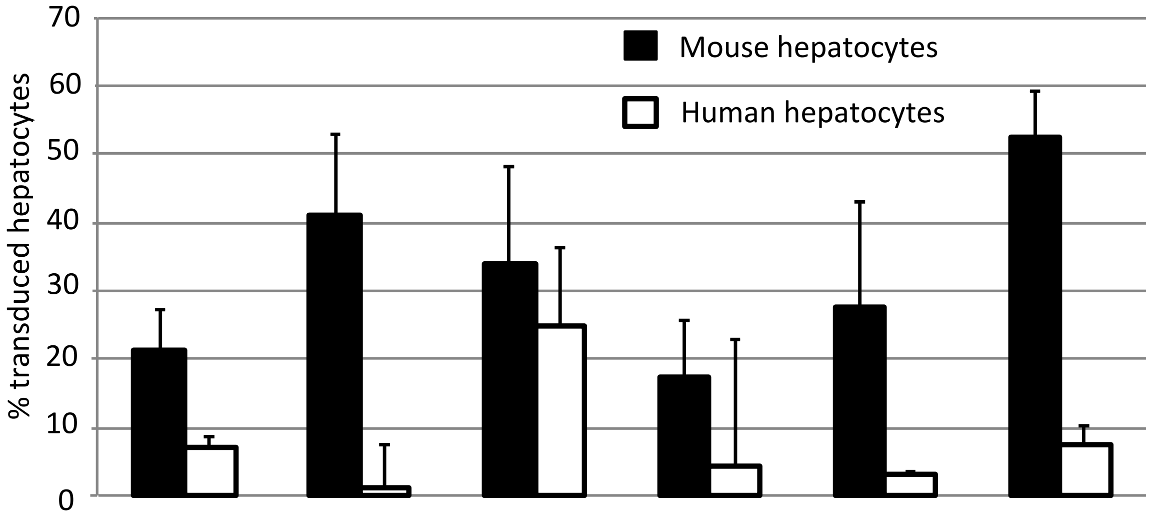

Capsid	8	8	9	9	9	9
Promoter	TBG	CB	CB	CB	TBG	CB
Reporter	LacZ	nLacZ	nLacZ	nLacZ	GFP	GFP
hALB	5.6	3.4	5.6	3.3	4.9	6.4
Mouse	1	3	2	4	5	6

Supplementary Figure 4. Quantification of AAV serotype transduction efficiencies in Ctr chimeric mice. Transduction of hepatocytes by intravenous tail vein injection of one (mouse #3-6) or two (mouse#1 and #2) AAV serotypes ($3x10^{11}$GC each). Different expression cassettes were used as depict. Quantification was done by counting LacZ- or GFP- positive human (white columns) and mouse (black columns) hepatocytes. Data shown is mean + standard deviation of 5-11 sets of images for each chimeric mouse liver . Human albumin levels (hALB) of each transduced mouse is given in mg/ml. TBG; thyroxin-binding globulin promoter, CB; CMV-enhanced chicken β-actin promoter.

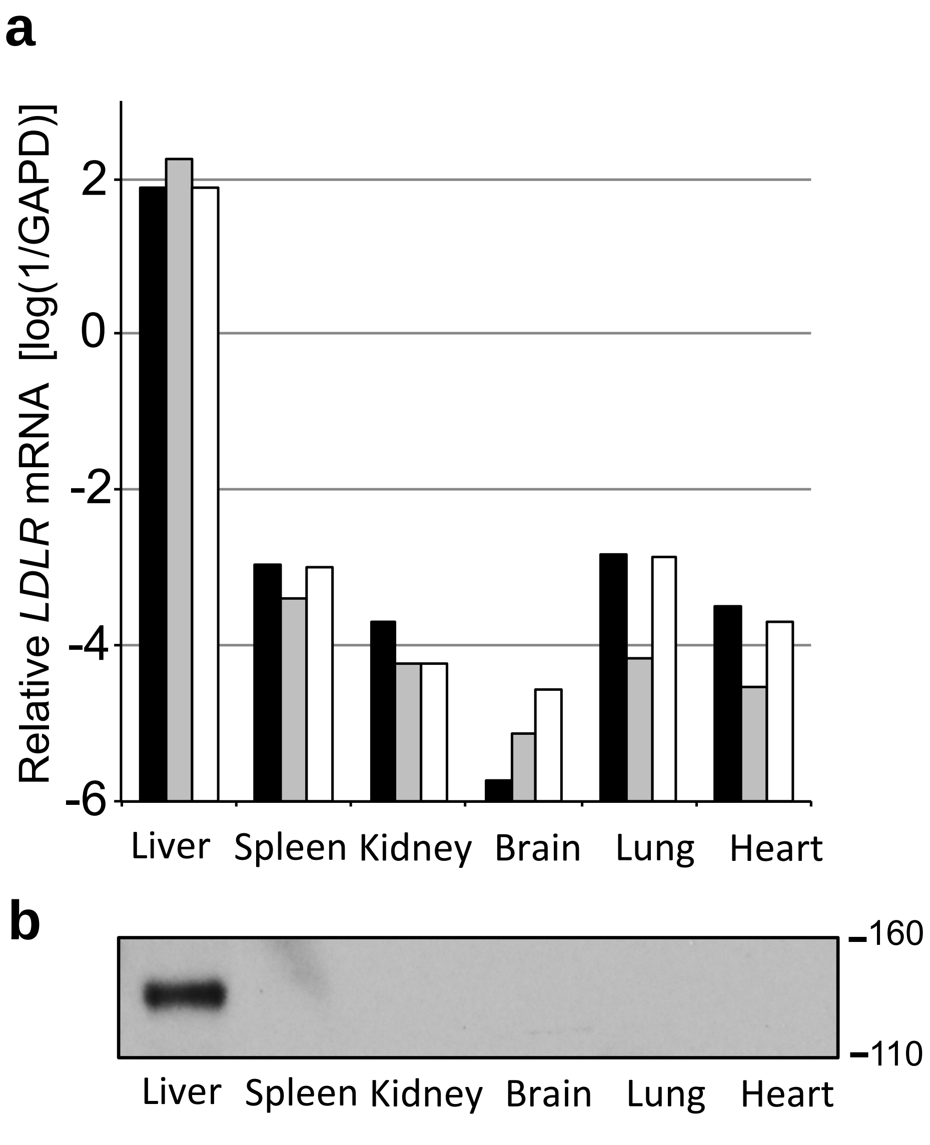

Supplementary Figure 5. Expression of LDLR in extrahepatic organs. (**a**), qRT-PCR for *hLDLR* mRNA of three AAV9-*LDLR* treated FH chimeric mice and (**b**), Western blotting with hLDLR antibodies using spleen, kidney, brain, lung, heart and liver (control) homogenate from a representative AAV9-*LDLR* treated FH chimeric mouse.

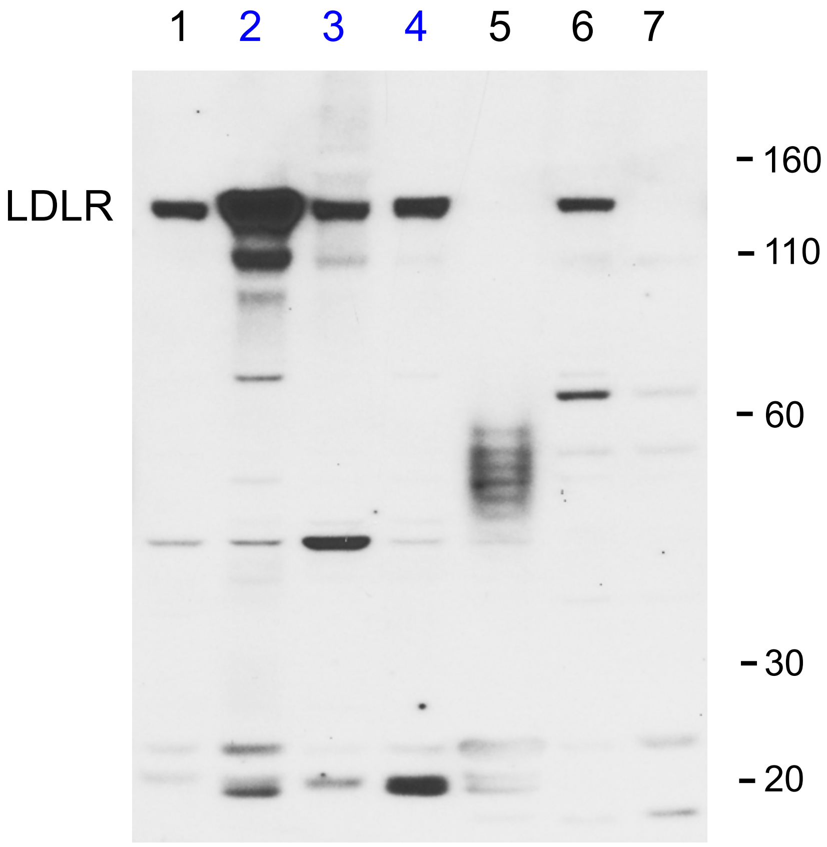

1. Ctr Heps → mouse
2. FH Heps → mouse AAV9-*LDLR*
3. FH Heps → mouse AAV9-*LDLR*
4. FH Heps → mouse AAV9-*LDLR*
5. FH Heps → mouse
6. Human Ctr Liver
7. Human FH Liver

Supplementary Figure 6. Expression of LDLR in liver homogenate of treated FH chimeric animals. Western blot reveals human LDLR in treated FH chimeric mice (blue) and control groups, demonstrating rescue of genetic defect underlying FH.

**Date (age/event)**	**Total Cholesterol** (normal: 135-200 mg/dL)	**LDL Cholesterol** (normal 60-140 mg/dL)	**HDL Cholesterol** (normal 35-73 mg/dL)	**Triglycerides** (normal: 20-150 mg/dL)	**Lipid-lowering medication**
5/2008 (3 years / diagnosis)	921	>350	32	152	none
7/2008	850	790	32	140	atorvastatin
9/2008	858	>350	35	128	
12/2008	831	>350	26	115	atorvastatin + ezetimibe
5/2009	954	>350	32	140	
7/2010	924	863	37	121	
5/2011	>1000	>350	32	90	
10/2011	820	>350	29	75	
8/2012	826	776	34	82	
11/2012* (7.5 years old / transplantation)	919	866	32	104	
11/12/2012	460	397	35	139	atorvastatin
12/2012	319	240	57	110	
3/2013	138	64	38	184	
10/2013	143	66	53	123	

Supplementary Table 1: Plasma lipid analysis and medication of patient with familial hypercholesterolemia. The first lipid analysis was performed shortly before diagnosis since the FH patient presented with xanthomas under her eye for several months (05/2008). Patient was put on lipid lowering medications as indicated, but plasma lipid profile remained pathological. *Indicates the time when the girl had liver transplantation.

Nucleotide	Zygosity	Amino Acid	Location	PolyPhen Prediction	Relationship to FH	References	Notes
c.401G>A	Het	p.Cys134Tyr	LDL Receptor Class A domain 3<br><br>Cys134 predicted to form disulfide bond with Cys116	Probably Damaging	Dominant, likely causative	Bertolini S. et al. [1]	p.Cys134Trp and pCys134Phe variants reported
c.1060+10G>C	Het	NA	Intronic	N/A; silent	Possibly associated	Amsellem S. et al. [2]<br><br>Dedoussis G.V. et al. [3]<br>Sozen M.M. et al. [4]	Not predicted to alter splicing
c.1103G>A	Het	p.Cys368Tyr	EGF-like Calcium binding domain 2<br><br>Cys368 predicted to form disulfide bond with Cys358	Probably Damaging	Dominant, likely causative	Loux N. et al. [5]<br><br>Mozas P. et al. [6]<br>Robles-Osorio L. et al. [7]<br>Sozen M.M. et al. [4]<br>Couture P. et al. [8]	Same variant<br><br>Same variant<br>Same variant<br>p.Cys368Gly variant reported<br>p.Cys368Arg variant reported
c.1413A>G	Het	p.Arg471Arg	LDL Receptor class B domain 2	N/A; silent	Not associated	Tatishcheva Iu A. et al. [9]	
c.1773C>T	Het	p.Asn591Asn	LDL receptor class B domain 5<br><br>Silent mutation in Beta Propellor Region,<br>alters splicing, mRNA stability and translation of LDLR	N/A; silent	Associated with LDL-C	Gao F. et al. [10]<br><br>Teslovich T.M. et al. [11]<br><br>Boright A.P. et al. [12]	rs688, common variant with MAF of 0.28<br><br>Associated with LDL-C in GWAS<br><br>4-10% increase depending on population
c.2232A>G	Het	p.Arg744Arg	Region with clustered O-linked oligosaccharides	N/A; silent	Not associated	Al-Khateeb A. et al. [13]	

Supplementary Table 2. Genetic analysis of *LDLR* locus.

Fraction	Accession	Coverage (%)	#Peptides	#Unique	Description
HDL	gi|114003	64	38	5	RecName: Full=Apolipoprotein A-II [Mus musculus]
HDL	gi|119573006	78	5	1	**apolipoprotein A-II_ isoform CRA_c [Homo sapiens]**
HDL	gi|12836446	5	3	3	unnamed protein product [Mus musculus]
HDL	gi|148694829	4	6	4	mCG142248 [Mus musculus]
HDL	gi|148706296	2	3	3	complement component 3_ isoform CRA_c [Mus musculus]
HDL	gi|157951676	84	57	10	apolipoprotein A-II precursor [Mus musculus]
HDL	gi|160333304	54	20	3	apolipoprotein A-I preproprotein [Mus musculus]g
HDL	gi|163310958	7	2	2	Chain A_ Structure Of Antiplasmin
HDL	gi|178775	10	2	1	**proapolipoprotein_ partial; ApoA-I [Homo sapiens]**
HDL	gi|18252782	5	2	2	antithrombin-III precursor [Mus musculus]
HDL	gi|186972736	61	5	5	**Chain A_ Structure And Dynamics Of Human Apolipoprotein C-Iii**
HDL	gi|191885	12	7	7	apolipoprotein A-IV [Mus musculus]
HDL	gi|199618	3	4	2	sex-limited protein [Mus musculus]
HDL	gi|199889	4	4	2	unnamed protein product [Mus musculus]
HDL	gi|2326168	1	2	2	type VII collagen [Mus musculus]
HDL	gi|26341396	7	4	4	unnamed protein product [Mus musculus]
HDL	gi|31982171	9	9	7	murinoglobulin-1 precursor [Mus musculus]
HDL	gi|387101	77	37	1	apolipoprotein A-II [Mus musculus]
HDL	gi|50017	84	44	1	apolipoprotein A-II [Mus musculus]
HDL	gi|61402210	33	11	1	Apoa1 protein [Mus musculus]
HDL	gi|619383	29	7	3	**apolipoprotein D_ apoD [human_ plasma_ Peptide_ 246 aa]**
HDL	gi|6680704	28	2	2	apolipoprotein C-I precursor [Mus musculus]
HDL	gi|6753100	24	2	2	apolipoprotein C-II precursor [Mus musculus]
HDL	gi|74146260	22	4	4	unnamed protein product [Mus musculus]
HDL	gi|8850219	6	2	2	haptoglobin precursor [Mus musculus]
LDL	gi|148683476	3	2	2	fibrinogen_ alpha polypeptide_ isoform CRA_a [Mus musculus]
LDL	gi|157951676	76	10	10	apolipoprotein A-II precursor [Mus musculus]
LDL	gi|163644329	25	9	6	apolipoprotein E precursor [Mus musculus]
LDL	gi|17016967	0	2	2	**NUANCE [Homo sapiens]**
LDL	gi|199086	3	7	7	alpha-2-macroglobulin [Mus musculus]
LDL	gi|342350764	13	3	1	**Chain A_ Nmr Structure Of Full Length Apoe3**
LDL	gi|6680704	28	3	3	apolipoprotein C-I precursor [Mus musculus]

Supplementary Table 3. Human and mouse proteins identified in lipoprotein fractions by mass spectrometry. HDL and LDL fractions were obtained from size-exclusion chromatography of plasma from a FH chimeric mouse. The peak fraction were delipidated, digested with trypsin, and subjected to proteomics by LC-MS. Proteins were identified based on a minimum of two different peptides matching an accession number for the respective human or mouse protein. The “Unique” column indicates the number of peptides for a given protein that is specific to either the mouse or human sequence respectively. Human proteins identified are indicated in bold.

Supplementary References

1. Bertolini S, *et al.* Clinical expression of familial hypercholesterolemia in clusters of mutations of the LDL receptor gene that cause a receptor-defective or receptor-negative phenotype. *Arterioscler Thromb Vasc Biol* **20**, E41-52 (2000).

2. Amsellem S, *et al.* Intronic mutations outside of Alu-repeat-rich domains of the LDL receptor gene are a cause of familial hypercholesterolemia. *Hum Genet* **111**, 501-510 (2002).

3. Dedoussis GV, *et al.* Molecular characterization of familial hypercholesterolemia in German and Greek patients. *Human mutation* **23**, 285-286 (2004).

4. Sozen MM, *et al.* The molecular basis of familial hypercholesterolaemia in Turkish patients. *Atherosclerosis* **180**, 63-71 (2005).

5. Loux N, *et al.* Screening for new mutations in the LDL receptor gene in seven French familial hypercholesterolemia families by the single strand conformation polymorphism method. *Human mutation* **1**, 325-332 (1992).

6. Mozas P, *et al.* Molecular characterization of familial hypercholesterolemia in Spain: identification of 39 novel and 77 recurrent mutations in LDLR. *Human mutation* **24**, 187 (2004).

7. Robles-Osorio L, *et al.* Genetic heterogeneity of autosomal dominant hypercholesterolemia in Mexico. *Arch Med Res* **37**, 102-108 (2006).

8. Couture P, *et al.* Identification of three mutations in the low-density lipoprotein receptor gene causing familial hypercholesterolemia among French Canadians. *Human mutation* **Suppl 1**, S226-231 (1998).

9. Tatishcheva Iu A, Mandel'shtam M, Golubkov VI, Lipovetskii BM, Gaitskhoki VS. [Four new mutations and polymorphic variants of the low density lipoprotein receptor in patients with familial hypercholesterolemia in Saint Petersburg]. *Genetika* **37**, 1290-1295 (2001).

10. Gao F, Ihn HE, Medina MW, Krauss RM. A common polymorphism in the LDL receptor gene has multiple effects on LDL receptor function. *Hum Mol Genet* **22**, 1424-1431 (2013).

11. Teslovich TM, *et al.* Biological, clinical and population relevance of 95 loci for blood lipids. *Nature* **466**, 707-713 (2010).

12. Boright AP, Connelly PW, Brunt JH, Morgan K, Hegele RA. Association and linkage of LDLR gene variation with variation in plasma low density lipoprotein cholesterol. *J Hum Genet* **43**, 153-159 (1998).

13. Al-Khateeb A, *et al.* Analysis of sequence variations in low-density lipoprotein receptor gene among Malaysian patients with familial hypercholesterolemia. *BMC Med Genet* **12**, 40 (2011).
